# Percutaneous nephrolithotomy versus retrograde intrarenal surgery for the treatment of kidney stones up to 2 cm in patients with solitary kidney: a single centre experience

**DOI:** 10.1186/s12894-017-0200-z

**Published:** 2017-01-18

**Authors:** Yunjin Bai, Xiaoming Wang, Yubo Yang, Ping Han, Jia Wang

**Affiliations:** Department of Urology, Institute of Urology, West China Hospital, Sichuan University, Chengdu, China

**Keywords:** Solitary kidney, Retrograde intrarenal surgery, Percutaneous nephrolithotomy

## Abstract

**Background:**

To compare the treatment outcomes between percutaneous nephrolithotomy (PCNL) and retrograde intrarenal surgery (RIRS) for the management of stones larger than 2 cm in patients with solitary kidney.

**Methods:**

One hundred sixteen patients with a solitary kidney who underwent RIRS (*n* = 56) or PCNL (*n* = 60) for large renal stones (>2 cm) between Jan 2010 and Nov 2015 have been considered. The patients’ characteristics, stone characteristics, operative time, incidence of complications, hospital stay, and stone-free rates (SFR) have been evaluated.

**Results:**

SFRs after one session were 19.6% and 35.7% for RIRS and PCNL respectively (*p* = 0.047), but the SFR at 3 months follow-up comparable in both groups (82.1% vs. 88.3%, *p* = 0.346). The calculated mean operative time for RIRS was longer (*p* < 0.001), but the mean postoperatively hospital stay was statistically significantly shorter (*p* < 0.001) and average drop in hemoglobin level was less (*p* = 0.040). PCNL showed a higher complication rate, although this difference was not statistically significant.

**Conclusions:**

Satisfactory stone clearance can be achieved with multi-session RIRS in the treatment of renal stones larger than 2 cm in patients with solitary kidney. RIRS can be considered as an alternative to PCNL in selected cases.

## Background

Renal calculi, especially large stone, are very dangerous for patients with solitary kidney. They may cause urinary tract infection, anuria, renal insufficiency or sepsis [1]. Therefore, stones in patients with solitary kidney need active treatment. The management of stones in this cohort as yet remains a challenging scenario, complete removal of the stone and protection of the renal function through safely surgical treatments is critical [1, 2].

Percutaneous nephrolithotomy (PCNL) is the mainstay of management for large (> 2 cm) or complicated renal stones [3]. Although this technique affords high success rates and accelerated stone clearance, regardless of stone composition and size [4], it is an aggressive treatment with severe complications for patients with solitary kidney. These patients are likely to have increased thickness of the renal parenchyma as a consequence of the compensatory hypertrophy, thus they are more likely to suffer bleeding when be treated with PCNL than patients with bilateral kidneys [5]. In addition, significant bleeding in these patients means potential acute renal failure due to urinary obstruction by blood clots and the absence of supplementary renal function of the other kidney [6]. Perhaps anatomically oriented access can be made so that the risk of this complication is minimized, but cannot be totally avoided.

In the past few years, improvements in endoscopy technology make retrograde intrarenal surgery (RIRS) more attractive, even for special circumstances, which has been used as an alternative option to PCNL for renal stones with a low complication rate [3]. In patients contraindicated for PCNL and with unfavorable treatment characteristics, such as morbid obesity, advanced vertebral deformities, serious cardiopulmonary diseases or those receiving anticoagulant treatment, RIRS is a reliable choice [3]. Which is a preferable treatment method for preserving functioning renal parenchyma [2], and this is crucial to the management of patients with solitary kidney [1]. Unfortunately, RIRS cannot be recommended as first-line treatment due to which stone-free rate (SFR) showed a negative correlation with stone size [7]. SFR after RIRS was achieved in 30% of patients with >2 cm stones and usually needed re-treatment; however, overall complication rates not related to stone sizes [7]. Therefore, patients with >2 cm stones should be counseled individually as staged procedures often required to remove calculi from the kidney without compromising the safety of RIRS. In addition, one concern about performing RIRS in a solitary kidney is the risk of renal function injury. Recently, Kuroda and coworkers [1] have shown that no significant difference was found in term of the change in glomerular filtration rate after RIRS between patients with solitary kidney and bilateral kidneys.

Current guidelines do not provide clear recommendations concerning the management of renal stones in patients with solitary kidney. Selecting the optimal management strategies for this cohort can be challenging, as each treatment modality has unique advantages and disadvantages. In the present study, we compared the efficacy and safety features between PCNL and RIRS with a flexible ureteroscope in the treatment of > 2 cm renal stones in patients with solitary kidney.

## Methods

After approval was obtained from the Institutional Review Board, the data of 116 consecutive patients with solitary kidney underwent PCNL or RIRS with a flexible ureteroscopy for kidney stones between January 2010 and November 2015 at our institution were retrospectively reviewed. Solitary kidney is identified as patients with either functional or anatomical solitary kidney. Solitary functional kidney is defined as patients whose preoperative evaluation showed a contralateral kidney function is < 5% in split renal function on a 99mTc-labeled dimercaptosuccinic acid single-photon emission computed tomography or drip infusion pyelography showed the contralateral kidney was significantly atrophic and had no urine secretion. The decision to perform PCNL or flexible ureteroscopy was based on individual surgeon discretion and patient selection.

Patient assessment before surgery included history-taking, clinical examination, laboratory examination, ultrasonography, plain radiograph of kidney-ureter-bladder (KUB), and non-contrast computed tomography (CT). Grade of hydronephrosis was categorized as none, mild, moderate, or severe, based on the appearance of the pelvis on ultrasonography and the presence of calices and/or parenchymal atrophy. Stone size was measured preoperatively and calculated as the sum of the largest axis of each stone on CT.

The operation time was defined as the time from the start of the first procedure to the termination of the surgical operation. For PCNL and RIRS, it was started with the puncture for an access tract and placement of flexible ureteroscope, respectively. The duration of hospitalization was defined as the time from the day of surgery to discharge for each session. Stone-free status was assessed by ultrasonography and/or a KUB, and was defined as the absence of any stones. Complications were classified using the Clavien-Dindo classification system [8].

## PCNL technique

Under general anesthesia and prone position, an 18 gauge needle was placed into proper calyx under C-arm fluoroscopy guidance. After a guidewire was inserted and fixed, dilation was performed serially with a fascial dilator up to 24 F and a 26 F sheath was placed through the tract. With using 8/9.8 F rigid ureteroscope, stone disintegration was performed using holmium laser and fragments were removed by flushing or forceps. An 18 F nephrostomy tube was placed at the end of the operation in all cases and usually removed on the fourth day after surgery, provided that there was no complication or the nephrostomy tube is draining clear urine.

## RIRS technique

Generally, a 6 F ureteral stent was placed 10–14 days before RIRS to relieve acute obstruction and infection, or to dilate the ureter for passage of the ureteroscope. Under general anesthesia, patients were positioned in lithotomy position. After two guidewires were advanced to the renal pelvis, a ureteral access sheath was implanted and a 7.5F flexible ureteroscope was inserted along the guidewires. Fragmentation of the stone burden was accomplished with a 4–12 W Holmium laser and then removed using stone basket. If operative time exceeded 90 min, we discontinued the procedure to minimize perioperative complications. At the end of the operation, a double-J stent was implanted in the pelvis routinely. KUB was taken on the first day after RIRS to assessed the residual stones and the location of the stents. Patients were reevaluated on the first and third postoperative month with laboratory examination, and KUB or CT scan. The double-J stent was removed under local anesthesia, as appropriate.

## Statistical method

The SPSS 19.0 software was used for all data analyses. Categorical variables were presented as number of subjects (n) and percentage (%), and analyzed using the Chi-squared or Fisher’s exact test as appropriate. The continuous data were presented as mean ± standard deviation and analyzed using the independent samples *t* test of variance. A two-sided *p* < 0.05 was considered to be statistically significant.

## Results

Patients’ characteristics and stone parameters are listed in Table 1. The groups were similar at baseline in terms of age, sex ratio, size and location distribution of stones, etiologies of the solitary kidney, comorbidities, and prevalence and grade of hydronephrosis (Table 1). Nineteen patients in PCNL and 55 in RIRS group were received double-J stent placement before surgery. Preoperative stenting and nephrostomy were carried out in 12 cases because of pyelonephritis in PCNL group. In RIRS group, a ureteral stent had been placed preoperatively to relieve acute obstruction and infection, or to dilate the ureter for passage of the ureteroscope.

**Table 1:** Clinical data of patients in PCNL and RIRS groups

	PCNL (*n* = 60)	RIRS (*n* = 56)	*P*
Age, yr	52.22 ± 10.56	48.84 ± 11.27	0.098
Gender, *n* (%)			0.395
Male	44 (73.3)	37 (66.1)	
Female	16 (26.7)	19 (33.9)	
Laterality, left, *n* (%)	33 (55.0)	27 (48.2)	0.465
Stone size, mm(range)	29.6 ± 5.7 (20–44)	27.7 ± 4.7(20–39)	0.052
Site of stone, *n* (%)			0.438
Pelvis	10 (16.7)	17 (30.4)	
Lower calyx	15 (25.0)	12 (21.4)	
Middle calyx	1 (1.7)	2 (3.6)	
Upper calyx	1 (1.7)	1 (1.8)	
Multiple	33 (55.0)	24 (42.9)	
Hydronephrosis, *n* (%)			0.054
None or mild	29 (48.3)	37 (66.1)	
Moderate or severe	31 (51.7)	19 (33.9)	
Preoperative double-J stent, *n* (%)	9 (15.0)	55 (98.2)	<0.001
Preoperative nephrostomy, *n* (%)	3 (5.0)	0	-
Recurrent stone former, *n* (%)	26 (43.3)	30 (53.6)	0.270
Comorbidities, n(%)			
Diabetes mellitus	9 (15.0)	7 (12.5)	0.696
Hypertension	13 (21.7)	9 (16.1)	0.442
Heart diseases	4 (6.7)	2 (3.6)	0.739
Renal insufficiency	13 (21.7)	9 (16.1)	0.442
Etiology of solitary kidney, *n* (%)			0.764
Contralateral nephrectomy	26 (43.3)	28 (50.0)	
Congenital	1 (1.7)	1 (1.8)	
Functional	33 (55.0)	27 (48.2)	
Stone composition, *n* (%)			0.307
Calcium based	45 (75.0)	39 (69.6)	
Uric acid	6 (10.0)	11 (19.6)	
Infection	9 (15.0)	6 (10.7)	

Perioperative and postoperative variables are presented in Table 2. The operation time in the RIRS group (99.46 ± 31.08 min) was significantly longer (*p* < 0.001) than that in the PCNL group (78.95 ± 29.81min), and a substantial number of patients with RIRS required reoperation. The postoperative hospital stay was significantly longer in PCNL group (*p* < 0.001). Kidney function as evaluated by serum creatinine level was stable for both approaches.

**Table 2:** Perioperative and Postoperative Data

	PCNL (*n* = 60)	RIRS (*n* = 56)	*P*
Operation time, min (range)^a^	78.75 ± 27.0 (42–141)	99.1 ± 29.5 (45–157)	<0.001
Postoperative hospitalization time, d (range)^a^	5.9 ± 1.5 (4–9)	2.0 ± 1.0 (1–5)	<0.001
Drop in Hb level in g/dl (range)	13.3 ± 6.6 (1.1–37.4)	10.2 ± 4.4 (2.8–21.3)	0.004
Initial stone-free, *n* (%)	25 (35.7)	11 (19.6)	0.047
Auxiliary procedures, *n* (%)			
PCNL	6 (10.0)	2 (3.6)	0.318
RIRS	12 (20.0)	27 (48.2)	0.001
Shock wave lithotripsy	7 (11.7)	19 (33.9)	0.004
Final stone-free rate, %	53 (88.3)	46 (82.1)	0.346
Preoperative serum creatinine in umol/L (range)	110.6 ± 38.1 (40.5–212.9)	113.8 ± 44.5 (18–263.4)	0.675
Postoperative serum creatinine in umol/L (range)	131.7 ± 57.4 (28.4–308.7)	136.6 ± 56.8 (28.8–305.5)	0.647
Complications (Clavein classification), %	19 (31.7)	14 (25.0)	0.426
Fever (G I)(%)	7 (11.7)	9 (16.1)	0.492
Urinary tract infection requiring additional antibiotics (G II) (%)	3 (5.0)	2 (3.6)	1.000
Urine leakage < 12 h(G II) (%)	1 (1.7)	0	-
Transfusion (G II) (%)	7 (11.7)	0	-
Steinstrasse (G IIIa) (%)	0	2 (3.6)	-
Sepsis (G IVa) (%)	1 (1.7)	1 (1.8)	1.000

^a^initial procedure plus auxiliary procedure

The initial SFR were 19.6% and 35.7% of the RIRS and PCNL groups, respectively (*p* = 0.047). Among patients with residual stones, 6 patients required second PCNL and 12 patients required RIRS in the PCNL group. In RIRS group 2 patients required PCNL, 27 patients required second RIRS. Other auxiliary procedures (shock wave lithotripsy, SWL) included 7 (11.7%) patients in PCNL group and 19 (33.9%) in RIRS group. After the auxiliary treatments, the final SFR at 3 months follow-up increased to 88.3% for PCNL group and 82.1% for RIRS group (*p* = 0.346).

Complications in both approaches are displayed in Table 2. The majority complications were graded I and II. Overall complication rate in the PCNL group was higher (31.7% vs. 25% in the PCNL and RIRS groups, respectively; *p* = 0.426). The infectious-related complications including fever and urinary tract infection requiring additional antibiotics were comparable between the two groups. Every group had one patient developed sepsis. The mean drop in the postoperative hemoglobin concentration in PCNL group was significantly higher than that in RIRS group (*p* = 0.004), and blood transfusions were required in 7 (11.7%) patients in the PCNL group. No nephrectomy or angioembolization was required. There was no significant difference between the two groups in stone compositions (*p* = 0.307).

## Discussion

Nowadays, the surgical management of renal stones has been dramatically changed because of tremendous reformation in endoscopy technology. As increased risk of perioperative complications and impairment of renal function for patients with solitary kidney during surgical management [6], thus, which surgical approach use continues to be of significant concern. In the era of minimally invasive surgery, RIRS and PCNL are two major surgical techniques for removing large renal stones [3], and PCNL has become the standard treatment with which all other approaches should be compared. A number of pertinent questions remain without conclusive answers, despite various studies reported in the literature, such as: how safe are PCNL or RIRS? What are the factors that portend a poor outcome with PCNL? How do complications compare PCNL with RIRS? Our results suggested that both PCNL and RIRS can safely be carried out for patients with solitary kidney. Final SFRs were similar in both groups. The main advantage of the RIRS over PCNL seems to be the less of mean decrease in the hemoglobin level. However, RIRS often required auxiliary treatment.

The primary concern of PCNL in solitary kidneys was the risk to develop complication such as severe uncontrollable bleeding that may cause an anephric state. The over complications after PCNL in these patients was 30.6%, of which 5.6% required blood transfusion [9]. Risk factors for serious bleeding include upper calix puncture, large stone, multiple tracts, inexperienced surgeon, and solitary kidney [5]. It was reported that the need for blood transfusion and the risk of severe bleeding were higher after PCNL in solitary kidneys compared to bilateral kidneys [5]. Hosseini and colleagues performed PCNL on 412 patients with solitary kidney, 19 (4.6%) patients encountered bleeding requiring transfusion, but none of them required nephrectomy [10]. Compensatory hypertrophy is common in solitary kidneys with increasing thickness of the renal parenchyma. It was speculated that access through such thick renal parenchyma may increase the risk of bleeding [5].

Continuous improvements in instruments and techniques of PCNL have helped urologists to perform this procedure with high levels of safety and efficacy in challenging cases such as stones in solitary kidneys [10]. Previous study reported that PCNL is a safe and efficient treatment for patients with solitary kidney despite the lower SFR (82.1% vs. 83.5%; *p* = 0.970) and increased morbidity (21.5% vs. 17.3%; *p* = 0.287) compared to patients with bilateral kidneys [11]. A recent systematic review confirmed the efficacy of PCNL for stones in patients with solitary kidney with initial and overall SFRs of 78.1% and 86.8% respectively [9]. It is surprising that PCNL for renal stones in these patients provided significant improvement in renal function [12]. In another study, Zeng and colleagues [2] compared the treatment outcomes between minimally invasive PCNL and RIRS for stones larger than 2 cm in patients with solitary kidney. They found SFRs after a single procedure were 71.7% in the minimally invasive PCNL group and 43.4% in the RIRS group (*p* = 0.003), and both groups with similar complications rates. Our single-session SFR in both groups was relatively low (35.7% vs 19.6% in the PCNL and RIRS groups, respectively). This may be related with that majority patients in our center had more complicated stones. In addition, the main reason for PCNL had a higher initial SFR than RIRS is that larger fragments fall back to the lower calix during RIRS.

Although SFR of RIRS is inferior to that of PCNL [13], considering patients with solitary kidney have the potential to encounter serious systemic disease, RIRS should always be considered at any time due to its efficacy and minimally invasive. Good outcomes of RIRS in terms of morbidity rate may be outweighed by its SFR in some cases, which is not neglected, especially in patients with solitary kidney. Bryniarski et al. [14] assessed outcomes after RIRS and PCNL. They found that transfusion required in 13 of PCNL patients and no transfusion in the RIRS patients. Gao et al.[15] have reported 26.6% (12/45) patients of RIRS encountered complications and 20% (9/45) were identified as I Clavien grade and no patients required blood transfusions. For our study, no major complications occurred and minor complications often were experienced. In our series, a 6 F stent had been routinely placed 10–14 days before RIRS to relieve acute obstruction and infection, which may be account for the infectious complications were also comparable between the two groups.

RIRS has been frequently considered in the treatment of larger renal stones as an alternative to PCNL. Although hemorrhagic diseases are often regarded as contraindications for both PCNL and SWL, RIRS demonstrated pretty safety in these patients [16]. Furthermore, with the increasing numbers of obese and morbid obese patients, the status of PCNL for renal stones may face challenges because great skin-kidney distance in these patients may lead to the puncture needle cannot reach the kidney. Fortunately, RIRS can be executed without limited outcomes for obese patients [17].

Stones in solitary kidney represent a management dilemma for the urologists. PCNL and RIRS are widely known to decrease surgery-related morbidity, while complete removal of calculi in solitary kidney from a single percutaneous or nature tract was difficult. Zhong et al [18] reported that combined use the two techniques can extract the calculi quickly, shorten operation time, make a high SFR. In addition, combined therapy can reduce the need for the number of tracts and then reduce the loss of blood and potential complications related to multiple tracts. Therefore, combined therapy can be used as a feasible treatment option for large renal stones in patients with solitary kidney.

RIRS is often performed as an ambulatory surgery in the Western countries. For patients and hospitals, they will choose RIRS as it is a less invasive treatment with less length of hospital stay. Under the culture background and the health insurance policy in China, both PCNL and RIRS were done as inpatient surgical procedure. Our patients are usually unwilling to discharge with the nephrostomy tube in place, thus, the hospital stay was longer in the both groups in our country. In addition, the solitary kidney patients in our series with large stones, treatment should be more careful and post-operative observation period needs to be extended. Our results are in line with other researches on RIRS or PCNL for large stone in China in term of hospitalization time [2, 15].

Our study has several limitations. First, this study was a retrospective design undertaken at a single center with a limit number of patients, we cannot eliminate the potential selection bias. Additionally, PCNL or RIRS in solitary kidney is a relative uncommon surgery and prospective design is challenging to be performed. Furthermore, the follow-up period of 3 months was quite short. We might not have detected the longer-term complications such as hypertension, renal impairment or ureteral stenosis.

## Conclusions

For larger than 2 cm renal stones in patients with solitary kidneys, PCNL offers initial SFRs superior to those of RIRS. However, satisfied outcomes can be acquired with multisession RIRS. Furthermore, hospital stay and complications of PCNL can be significantly reduced with RIRS. Therefore, RIRS represents a good alternative treatment to PCNL in well selected cases with larger renal stones in patients with solitary kidneys.

## Abbreviations

CCS: Case-control study
CI: Confidence interval
LPL: Laparoscopic pyelolithotomy
MD: Mean difference
NOS: Newcastle-Ottawa Scale
OR: Odds ratio
PCNL: Percutaneous nephrolithotomy
RCT: Randomized controlled trial
SFR: Stone-free rate
UTI: Urinary tract infection
